# Dendritic spine dysgenesis in Rett syndrome

**DOI:** 10.3389/fnana.2014.00097

**Published:** 2014-09-10

**Authors:** Xin Xu, Eric C. Miller, Lucas Pozzo-Miller

**Affiliations:** Department of Neurobiology, Civitan International Research Center, The University of Alabama at Birmingham, Birmingham, ALUSA

**Keywords:** MeCP2, BDNF, excitatory synapse, spine density, hippocampus, organotypic slice cultures, TrkB, autism spectrum disorder

## Abstract

Spines are small cytoplasmic extensions of dendrites that form the postsynaptic compartment of the majority of excitatory synapses in the mammalian brain. Alterations in the numerical density, size, and shape of dendritic spines have been correlated with neuronal dysfunction in several neurological and neurodevelopmental disorders associated with intellectual disability, including Rett syndrome (RTT). RTT is a progressive neurodevelopmental disorder associated with intellectual disability that is caused by loss of function mutations in the transcriptional regulator methyl CpG-binding protein 2 (*MECP2*). Here, we review the evidence demonstrating that principal neurons in RTT individuals and *Mecp2*-based experimental models exhibit alterations in the number and morphology of dendritic spines. We also discuss the exciting possibility that signaling pathways downstream of brain-derived neurotrophic factor (BDNF), which is transcriptionally regulated by MeCP2, offer promising therapeutic options for modulating dendritic spine development and plasticity in RTT and other *MECP2*-associated neurodevelopmental disorders.

## INTRODUCTION

*Espinas dendríticas* are small cytoplasmic extensions emerging from the dendrites of neurons that were first described in the cerebellum and cerebrum of birds and mammals by Santiago Ramón y Cajal at the end of the 19th century (Ramón y Cajal, 1888, 1891b, 1896; as cited in Yuste, 2010). Cajal had already envisioned that dendritic spines are contacted by axons at synapses (Ramón y Cajal, 1891a, 1894), and used this arrangement as the main example in support of his *Neuronal Doctrine* (Ramón y Cajal, 1933). With the aid of electron microscopy and confocal fluorescence microscopy, it is now well established that spines are the postsynaptic sites of most excitatory synapses in the brain, receiving inputs from glutamatergic axons (Bhatt et al., 2009; Yuste, 2010; Shirao and Gonzalez-Billault, 2013). Despite their small size (∼1 μm in diameter), proper dendritic spine formation is critical for brain function. Numerous proteins, including neurotransmitter and neuropeptide receptors, signaling kinases, and phosphatases, as well as ion channels are expressed in dendritic spines, where they participate in excitatory synaptic transmission and activity-dependent synaptic plasticity, and ultimately in learning and memory (Sala and Segal, 2014). During development and throughout adulthood, the numerical density and morphology of individual spines are critical for the fine-tuning of neuronal and synaptic excitability, allowing the initial establishment and activity-dependent remodeling of connectivity of neuronal circuits (Luebke et al., 2010).

The morphology of dendritic spines is highly variable, and by defining the biochemical and electrical properties of the postsynaptic compartment, it contributes to the strength and plasticity of excitatory synapses (Luebke et al., 2010). Spines have been broadly classified into three morphological types: stubby, mushroom and thin (Peters and Kaiserman-Abramof, 1969). Mushroom spines have a large head that is connected to the parent dendrite through a narrow neck. Stubby spines do not have a noticeable neck and are most common during postnatal development (Chapleau et al., 2009b; Rochefort and Konnerth, 2012). These two types of large spines are referred to as “memory spines,” because they are stable, persist for longer periods of time, and are the postsynaptic side of strong excitatory synapses (Trachtenberg et al., 2002; Kasai et al., 2003). Conversely, thin spines have a thin, long neck, and a small bulbous head, are highly motile, unstable, and often short-lived, usually representing weak or silent synapses (Rochefort and Konnerth, 2012). Because thin spines are more plastic than large spines and have the potential to become stable spines, they have been dubbed “learning spines” (Grutzendler et al., 2002; Trachtenberg et al., 2002; Kasai et al., 2003; Holtmaat et al., 2005). It should be noted that thin protrusions longer than thin spines and without a noticeable head are called dendritic filopodia, and are more numerous than spines in developing neurons. Dendritic filopodia are transient and highly motile protrusions that can receive synaptic input and develop into mature spines, thus initiating synaptogenesis (Fiala et al., 1998; Luebke et al., 2010).

Following the well-known relationship between form and function in biological systems, recent *in vitro* and *in vivo* studies have demonstrated that the morphology of spines relates closely to the function and plasticity of the synapses they belong to (Yuste et al., 2000; Trachtenberg et al., 2002; Mizrahi et al., 2004; Segal, 2005; Kasai et al., 2010). For example, the volume of the spine head is directly proportional to the area of the postsynaptic density and the number of synaptic vesicles docked at the presynaptic active zone (Harris and Stevens, 1989; Schikorski and Stevens, 1999), the number of postsynaptic receptors (Nusser et al., 1998), and hence to the size of synaptic currents and synaptic strength (Yuste and Bonhoeffer, 2001; Luebke et al., 2010). Two-photon uncaging of glutamate on large spines evoked larger postsynaptic currents mediated by AMPA receptors than uncaging on small spines (Matsuzaki et al., 2001; Kasai et al., 2010). Such a structure-function relationship is also evident in the intracellular Ca^2+^ signals within spines triggered by afferent synaptic activity (Yuste et al., 2000; Nimchinsky et al., 2002; Bloodgood and Sabatini, 2007). Together with structural changes in response to afferent synaptic stimulation (Murphy and Segal, 1996; Srivastava and Penzes, 2011), all these findings support the long held view that dendritic spines are the morphological substrate of neuronal plasticity and learning and memory (Sala and Segal, 2014). In support of this notion, induction of long-term potentiation (LTP) leads to spine enlargement (Matsuzaki et al., 2004; Park et al., 2006), whereas long-term depression (LTD) causes spine shrinkage (Nagerl et al., 2004; Zhou et al., 2004; Hoogenraad and Akhmanova, 2010; Penzes et al., 2011b).

The relationship between dendritic spines and cognitive abilities was noted in early studies, when the term “spine dysgenesis” was coined by Dominick Purpura (Huttenlocher, 1970; Marin-Padilla, 1972; Purpura, 1974). Such anomalies in the morphology – and likely function – of dendritic spines have been described in several neurological disorders associated with cognitive decline, including typical aging, Alzheimer’s and Huntington diseases, schizophrenia, neurodevelopmental intellectual disabilities, and autism spectrum disorders (Fiala et al., 2002; Fukuda et al., 2005; Zhao et al., 2007; Bourgeron, 2009; Chapleau et al., 2009b; Garey, 2010; Penzes et al., 2011a; Levenga and Willemsen, 2012).

## DENDRITIC SPINE DYSGENESIS IN RETT SYNDROME

Rett syndrome (RTT) is an X-linked progressive autism spectrum disorder associated with intellectual disability that affects girls during early childhood (∼1:15,000 birth worldwide; Neul and Zoghbi, 2004; Chapleau et al., 2009b). The disorder is characterized by a seemingly typical development for 6 to 18 months followed by regression and onset of a variety of neurological features, including motor impairments, loss of acquired language, intellectual disability, seizures, and anxiety (Chahrour and Zoghbi, 2007). The majority of RTT individuals carry loss-of-function mutations in *MECP2*, the gene encoding methyl CpG-binding protein 2 (MeCP2), a global transcriptional regulator that binds to methylated CpG sites in promoter regions of DNA (Amir et al., 1999; Chahrour et al., 2008). Emerging evidence indicates that RTT results from a deficit in synaptic maturation in the brain, and that MeCP2 plays a critical role in neuronal and synaptic maturation and pruning during development (Cohen et al., 2003; Calfa et al., 2011b), as well as in the function of established neuronal networks in adulthood (McGraw et al., 2011).

**FIGURE 1:**
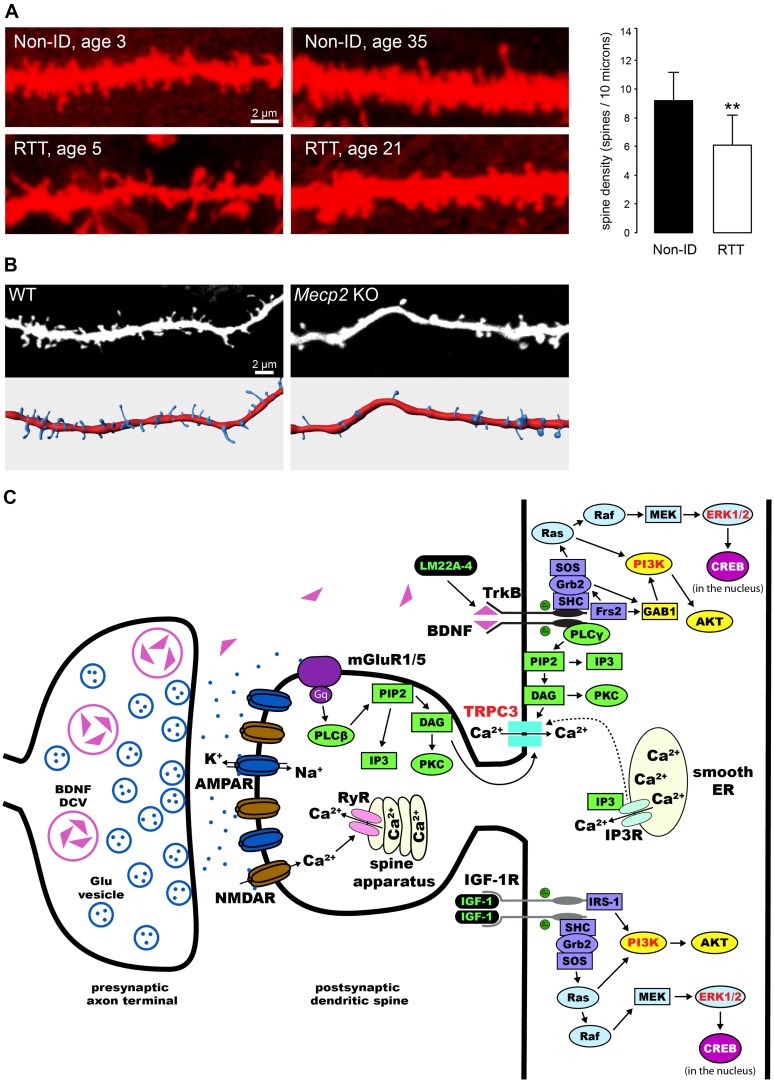
**Dendritic spine dysgenesis in Rett syndrome, and intracellular signaling cascades involved in spine plasticity mediated by BDNF and IGF-1. (A)** Confocal images of human CA1 pyramidal neurons in hippocampal sections from autopsy material labeled with DiI. Neurons from RTT individuals have lower dendritic spine density than those from typically developing individuals (Non-ID, non-intellectually disabled). **P < 0.01 (adapted from Chapleau et al., 2009a).** (B)** Confocal images of apical dendritic segments (top) of eYFP-expressing CA1 pyramidal neurons in 11 days *in vitro* hippocampal slice cultures prepared from postnatal day-5 wildtype (WT) and *Mecp*2 knockout (KO) mice, and their corresponding surface-rendered reconstructions (bottom).** (C)** Schematic diagram of an exemplary excitatory synapse on a dendritic spine of a pyramidal neuron in the hippocampus. We highlight the intracellular signaling cascades that mediate the effects of BDNF and IGF-1 on structural plasticity of spines. TrkB receptors are activated upon binding of BDNF, leading to dimerization and auto-phosphorylation. This process allows for the binding of adaptor proteins to their intracellular domain, and the subsequent activation of Ras/ERK, PI3K, and PLCγ (reviewed by Huang and Reichardt, 2003). All these pathways have been implicated in the effects of BDNF on dendritic spines (highlighted in red, see text for references). Potential therapies for the treatment of RTT act on these pathways (highlighted in green, see text for details and references): LM22A-4 binds and activates TrkB receptors directly (Massa et al., 2010); activation of IGF-1 receptors triggers the PI3K and Ras/ERK signaling pathways (Zheng and Quirion, 2004). DCV, dense core vesicle; Glu, glutamate; AMPAR, α-amino-3-hydroxy-5-methyl-4-isoxazolepropionic acid receptor; NMDAR, *N*-methyl-D-aspartate receptor; mGluR, metabotropic glutamate receptor; ER, endoplasmic reticulum; RyR, ryanodine receptor; PIP2, phosphatidylinositol 4,5 bisphosphate; DAG, diacylglycerol; IP3, inositol triphosphate; IP3R, IP3 receptor; PKC, protein kinase C; SH-2, src homology domain 2; SHC, SH-2-containing protein; Grb2, growth factor receptor-binding protein 2; GAB1, Grb2-associated-binding protein 1; SOS, nucleotide exchange factor *son-of-sevenless*; Frs2, fibroblast growth factor receptor substrate 2; AKT, protein kinase B; Ras, rat sarcoma proto-oncogenic G-protein; Raf, proto-oncogenic serine/threonine protein kinase; MAPK, mitogen-activated protein kinase; MEK, MAPK kinase; cAMP, cyclic adenosine monophosphate; CREB, cAMP response element-binding protein; IGF-1R, IGF-1 receptor; IRS-1, insulin receptor substrate 1.

Pyramidal neurons in the cortex and hippocampus of RTT individuals have dendrites with atypical morphology (Belichenko et al., 1994; Armstrong et al., 1995; Chapleau et al., 2009a; **Figure 1A**). Two different mouse models lacking *Mecp2* (Chen et al., 2001; Guy et al., 2001) have reduced dendritic complexity (Fukuda et al., 2005; Nguyen et al., 2012; Stuss et al., 2012), and decreased dendritic spine density and motility in cortical and hippocampal neurons (Belichenko et al., 2009; Tropea et al., 2009; Landi et al., 2011; Chapleau et al., 2012; Castro et al., 2014). On the other hand, *Mecp2^308^* mice expressing truncated MeCP2 (Shahbazian et al., 2002), which have impaired synaptic plasticity and hippocampal-dependent learning and memory and other RTT-related neurological deficits, do not show any dendritic or synaptic anomalies neither in cortical nor hippocampal neurons (Moretti et al., 2006). The reduced dendritic spine density, along with a decrease in the proportion of mushroom spines, is also present in primary hippocampal neurons (Chao et al., 2007; Baj et al., 2014) and hippocampal slice cultures (**Figure 1B**) prepared from newborn *Mecp2* knockout (KO) pups, as well as in neurons derived from induced pluripotent stem cells obtained from RTT individuals (Marchetto et al., 2010).

The spine dysgenesis phenotype in pyramidal neurons of the hippocampus in *Mecp2* KO mice has revealed unexpected complexities. CA1 and CA3 pyramidal neurons have lower spine density only in neonatal (postnatal day-7) *Mecp2* KOs, well before excitatory synapse expansion. Spine density reaches wildtype (WT) levels a week later (postnatal day-15), and is maintained at WT levels throughout the symptomatic stage (postnatal day-40 to 60). Quantitative electron microscopy confirmed that the density of asymmetric spine synapses in CA1 *stratum radiatum* of *Mecp2* KOs is comparable to that of WT mice (Calfa et al., 2011a; Chapleau et al., 2012). This developmental progression of the spine density phenotype is also reflected in the density of excitatory synapses imaged as VGLUT1-PSD95 immunofluorescent puncta, which is lower in area CA1 of 2 week-old *Mecp2* null mice, but comparable to WT levels at 5 weeks of age (Chao et al., 2007). Altogether, these data demonstrate that proper MeCP2 functioning is required for dendritic spine formation during early postnatal development, and that a secondary compensatory mechanism seems to take place during atypical development in *Mecp2* KOs. A couple of possibilities exist as to the extent of the compensatory mechanisms necessary to bring spine density to WT levels in hippocampal neurons. One possibility is that enhanced hippocampal network activity in *Mecp2* KOs promotes dendritic spine formation (Calfa et al., 2011a). A second possibility is that deranged homeostatic plasticity promotes spinogenesis, while still affecting pyramidal neuron function (Blackman et al., 2012; Qiu et al., 2012).

Consistent with a model that tightly regulated MeCP2 levels are necessary during brain development and adulthood, overexpression of *Mecp2 in vitro* or in the *MECP2* duplication mouse model (*Mecp2*^Tg1^) either increased (Jugloff et al., 2005; Chao et al., 2007; Jiang et al., 2013) or decreased (Zhou et al., 2006; Chapleau et al., 2009a; Cheng et al., 2014) dendritic complexity, spine density, and the density of excitatory synapses. **Table 1** sumarizes all the published work on dendritic spines in RTT and experimenal models based on MeCP2 loss-of-function.

**Table 1:** Dendritic spine dysgenesis in RTT individuals and MeCP2-deficient cells and mice.

Source	Brain region	Preparation	Alterations in dendrites and dendritic spines	Reference
RTT individuals	Cerebral cortex	Fixed postmortem brain (layer II and III at 2.9–35 years old)	↓Dendritic complexity ↓Dendritic spine density	Belichenko et al. (1994), Armstrong et al. (1995)
	Hippocampus	Fixed postmortem brain (CA1 region at 1–42 years old)	↓Dendritic spine density	Chapleau et al. (2009a)
	Induced pluripotent stem cells	Fibroblasts from patients’ dermal biopsies (DIV56)	↓Excitatory synapse number ↓Dendritic spine density	Marchetto et al. (2010)
*Mecp2*^tm.1.1Bird^	Cortex	Fixed brain (layer II/III motor cortex at P21)	↓Dendritic spine density	Belichenko et al. (2009)
		Fixed brain (layer II/III somatosensory cortex at P42)	↓Dendritic complexity ↓Dendritic spine density	Fukuda et al. (2005)
	Hippocampus	Autaptic culture (DIV7–9)	↓Excitatory synapse number	Chao et al. (2007)
		Primary culture (DIV9–15)	↓Excitatory synapse number ↓Dendritic complexity ↓Dendritic spine density ↓Mushroom spines ↑Stubby spines	Baj et al. (2014)
		Fixed brain (CA1 region at P21)	↓Dendritic spine density	Belichenko et al. (2009)
		Fixed brain (CA1 region at P42)	↓Dendritic complexity ↓Dendritic spine density	Nguyen et al. (2012)
	Fascia dentata	Fixed brain (P21)	↓Dendritic spine density	Belichenko et al. (2009)
*Mecp2*^tm.1.1Jae^	Cortex	*In vivo* and fixed brain (layer V somatosensory cortex at P25, P40)	↓Dendritic spine density altered spine dynamics	Landi et al. (2011)
		Fixed brain (layer V motor cortex at P40)	↓Dendritic complexity ↓Dendritic spine density	Stuss et al. (2012)
		Fixed brain (layer II/III visual cortex at P42)	↓Dendritic spine density	Castro et al. (2014)
		Fixed brain (layer V motor cortex at P60)	↓Dendritic spine density	Tropea et al. (2009)
	Hippocampus	Fixed brain (CA1 region at P7)	↓Dendritic spine density	Chapleau et al. (2012)
		Fixed brain (CA1 region at P21)	↓Dendritic spine density	Belichenko et al. (2009)
		Fixed brain (newly matured DG neurons at P56)	↓Dendritic spine density	Smrt et al. (2007)
*Mecp2* knockdown	Cortex	Primary culture (layer II/III visual cortex at DIV7-9)	↓Excitatory synapse number	Blackman et al. (2012)
	Hippocampus	Slice culture (CA1 region at DIV11)	↓Dendritic spine density ↓Mature spines	Chapleau et al. (2009a)
*Mecp2*^Tg1^	Cortex	*In vivo* (layer V somatosensory cortex at P56)	↑Dendritic spine density	Jiang et al. (2013)
	Hippocampus	Autaptic culture (DIV7–9)	↑Excitatory synapse number	Chao et al. (2007)
Overexpression of *MECP2*	Cortex	Primary culture (DIV6)	↑Dendritic complexity	Jugloff et al. (2005)
		Primary culture (DIV6)	↓Dendritic complexity	Cheng et al. (2014)
	Hippocampus	Slice culture (pyramidal neurons at DIV7)	↓Dendritic complexity ↑Thin spines	Zhou et al. (2006)
		Slice culture (pyramidal neurons at DIV9)	↓Dendritic complexity ↓Dendritic spine density	Cheng et al. (2014)
		Slice culture (CA1 region at DIV9)	↓Dendritic spine density ↓Mature spines	Chapleau et al. (2009a)
Overexpression of *MECP2* mutations (R106W and T158M)	Hippocampus	Slice culture (CA1 region at DIV9–11)	↓Dendritic spine density ↓Mature spines	Chapleau et al. (2009a)

## ROLE OF BDNF IN DENDRITIC SPINE FORMATION AND PLASTICITY: A POTENTIAL THERAPY FOR RTT

MeCP2 regulates the expression of thousands of genes, including brain-derived neurotrophic factor (*Bdnf*; Chen et al., 2003; Martinowich et al., 2003; Zhou et al., 2006). BDNF is well known to promote neuronal and synaptic maturation (Carvalho et al., 2008), increase dendritic spine density, and enhance synaptic plasticity and learning and memory (Figurov et al., 1996; Luine and Frankfurt, 2013). MeCP2 binds to the *Bdnf* promoter and modulates *Bdnf* expression in an activity-dependent manner (Chen et al., 2003; Martinowich et al., 2003; Zhou et al., 2006; Chahrour and Zoghbi, 2007). Lower *Bdnf* mRNA and BDNF protein levels, as well as impaired BDNF trafficking and activity-dependent release, have been highlighted as pathophysiological mechanisms of RTT disease progression (Chang et al., 2006; Wang et al., 2006; Ogier et al., 2007; Li et al., 2012; Xu et al., 2014). Indeed, overexpression of BDNF rescues several cellular and behavioral deficits in *Mecp2* KO mice (Chang et al., 2006; Chahrour and Zoghbi, 2007). These studies indicate that BDNF plays a critical role in neurological impairments in MeCP2-deficient mice.

Several studies have demonstrated that BDNF participates in synaptic plasticity, and is critical for dendritic spine formation and maturation during development (Poo, 2001; Tyler et al., 2002a; Tanaka et al., 2008; Cohen-Cory et al., 2010; Vigers et al., 2012). For example, exogenously applied BDNF increases spine density in cultured hippocampal neurons and CA1 pyramidal neurons in slice cultures (Tyler and Pozzo-Miller, 2001; Ji et al., 2005). In addition, BDNF shifts the proportions of morphological types of spines in hippocampal slice cultures (Tyler and Pozzo-Miller, 2003; Chapleau et al., 2008). Moreover, overexpression of the *Bdnf* gene in cultured hippocampal neurons rescued the dendritic atrophy caused by shRNA-mediated *Mecp2* knockdown (Larimore et al., 2009). These effects of BDNF on dendritic spines are mediated by the tropomyosin related kinase B (TrkB) receptor (Tyler and Pozzo-Miller, 2001), and subsequent activation of extracellular signal-regulated kinase (ERK; Alonso et al., 2004), phosphatidylinositol 3-kinase (PI3K; Kumar et al., 2005), and phospholipase C-γ (PLC-γ), which leads to the opening of canonical transient receptor potential (TRPC) channels containing the TRPC3 subunit (Amaral and Pozzo-Miller, 2007; Chapleau et al., 2009b; Li et al., 2012; Luine and Frankfurt, 2013; **Figure 1C**).

Activity-dependent release of endogenously expressed (native) BDNF also modulates spine morphology in conjunction with spontaneous neurotransmitter release (Tyler et al., 2002b; Tyler and Pozzo-Miller, 2003; Tanaka et al., 2008). In addition, proper secretory trafficking of BDNF is essential for its actions on dendritic spine development and plasticity. The human *BDNF* gene has a single nucleotide polymorphism – a methionine (met) substitution for valine (val) at codon 66 – that impairs BDNF trafficking and its activity-dependent release, resulting in cognitive dysfunction in the general population (Egan et al., 2003; Chen et al., 2004), as well as more severe neurological symptoms in RTT individuals (Zeev et al., 2009). Consistently, dendritic complexity is reduced in dentate granule cells of Val66Met knock-in mice (Chen et al., 2006). Therefore, expression of this *BDNF* polymorphism might lead to deleterious effects on dendritic spine density and morphology.

The main limitation of BDNF-based therapies for neurological disorders, including RTT, is its poor blood-brain barrier permeability. Synthetic BDNF-loop mimetics with selective TrkB agonist activity are exciting alternatives (Massa et al., 2010; Kajiya et al., 2014). Indeed, systemic treatment with LM22A-4 rescues respiratory deficits in female *Mecp2* heterozygous mice (Schmid et al., 2012), and prevents spine loss in striatal medium-spiny neurons in a mouse model of Huntington’s, rescuing their motor deficits (Simmons et al., 2013).

Other intriguing substitutes for BDNF are insulin-like growth factor-1 (IGF-1) and its active tripeptide ([1–3]IGF-1, also known as glypromate, GPE), a hormone widely expressed in the CNS during brain development that promotes neuronal survival as well as synaptic maturation (D’Ercole, 1996; O’Kusky et al., 2000; Tropea et al., 2009). Indeed, systemic treatment of male *Mecp*2 KO mice with [1–3]IGF-1 significantly increased activity of signaling pathways downstream of TrkB and improved several RTT-like symptoms and increased dendritic spine density in cortical neurons (Tropea et al., 2009), effects that are all recapitulated by full-length IGF-1 (Castro et al., 2014). These effects are due to the activation of IGF-1 receptors directly by IGF-1, and indirectly by [1–3]IGF-1, which does not bind to the IGF-1 receptor but rather increases the expression of IGF-1 (Carlsson-Skwirut et al., 1989; Corvin et al., 2012). It should be noted that full-length IGF-1 worsened a metabolic syndrome in *Mecp2* KO mice, and did not affect dendritic spine density in hippocampal neurons (Pitcher et al., 2013). The safety and efficacy of recombinant human full-length IGF-1 (mecasermin) in a Phase-1 clinical trial in RTT individuals have been recently reported (Khwaja et al., 2014). The [1–3]IGF-1 analog glycyl-L-methylprolyl-L-glutamic acid (NNZ-2566; Neuren Pharmaceuticals) is in a Phase-2 clinical trial in RTT individuals.

## CONCLUSION

Activity-dependent plasticity of dendritic spines includes both the formation of new spines and their maturation from thin, filipodia-like protrusions to “memory spines” that accompany excitatory synapse formation during brain development, as well as the structural remodeling of already existing spines. Alterations in neuronal circuitry are due to, or at least reflected by, deficits in dendritic spine structure and function. Dendritic spine anomalies have been identified in multiple brain regions in RTT and *Mecp2*-based mouse models. Since BDNF promotes the formation, maintenance, and activity-dependent sculpting of dendritic spines, and plays a critical role in neurological dysfunction in RTT, it emerges as one of the most exciting therapeutic agents for RTT. Thus, treatments that target the BDNF receptor TrkB and/or its downstream signaling pathways stand out as strong candidates to improve not only the spine dysgenesis phenotype, but also other synaptic plasticity deficits in RTT and other neurodevelopmental disorders caused by impaired BDNF availability.

## Conflict of Interest Statement

The authors declare that the research was conducted in the absence of any commercial or financial relationships that could be construed as a potential conflict of interest.
